# Anlotinib Combined With Chemoradiotherapy Exhibits Significant Therapeutic Efficacy in Esophageal Squamous Cell Carcinoma

**DOI:** 10.3389/fonc.2020.00995

**Published:** 2020-07-10

**Authors:** Jingzhen Shi, Yingjie Zhang, Jinzhi Wang, Jianbin Li, Zhenxiang Li

**Affiliations:** ^1^School of Medicine, Shandong University, Jinan, China; ^2^Shandong Cancer Hospital Affiliated to Shandong University, Jinan, China; ^3^Department of Radiation Oncology, Shandong Cancer Hospital and Institute, Shandong First Medical University and Shandong Academy of Medical Sciences, Jinan, China

**Keywords:** patient-derived xenograft, esophageal squamous cell carcinoma, anlotinib, chemoradiotherapy, anti-angiogenesis

## Abstract

**Objective:** The aim of this study was to evaluate the safety and efficacy of anlotinib combined with chemoradiotherapy for treating esophageal squamous cell carcinoma (ESCC) using patient-derived xenografts (PDXs).

**Methods:** PDX-bearing mice were randomly divided into five groups, as follows: control group receiving normal saline, the group receiving radiotherapy, the group receiving cisplatin combined with radiotherapy, the group receiving anlotinib combined with radiotherapy, and the group receiving anlotinib, and cisplatin combined with radiotherapy. Tumor volumes and body weights were measured three times weekly for 2 weeks. The PDXs were initially assessed by comparing the histology of the original patient tumor tissues with that of the corresponding serially passaged xenografts by hematoxylin and eosin (H&E) and P63 staining. Then, expression of Bax, c-PARP, PCNA, and CD31 was detected using immunohistochemistry, and apoptosis was detected by a TUNEL assay. Cytokines released into plasma were analyzed using protein chip technology. Finally, two case studies of ESCC patients were presented to further verify the results observed in the PDX models.

**Results:** The pathological characteristics of the serially passaged patient tumor-derived xenografts established in our study were in line with those of the original ESCC patient samples. The group receiving anlotinib and cisplatin plus radiotherapy exhibited the strongest antitumor response among the groups. Moreover, the ideal anticancer effects of anlotinib combined with chemoradiotherapy observed in clinical patients were consistent with the results observed in the PDX models, and no serious side effects were observed during treatment.

**Conclusions:** Combination therapy with anlotinib and chemoradiotherapy may be an effective regimen for the treatment of advanced ESCC.

## Introduction

According to the 2015 China Cancer Report (1), esophageal cancer (EC) is predicted to be the sixth most commonly diagnosed cancer (246,000 new cases) and the fourth leading cause of cancer death (188,000 deaths) in China. Esophageal squamous cell carcinoma (ESCC), accounting for more than 95% of all cases of EC, was deemed to the predominant histological type in China (1, 2). Patients usually present at advanced stages, and the 5-years overall survival (OS) rate of these patients is <5%; treatment options traditionally include surgery, chemotherapy, and radiotherapy (3). Recently, advanced esophageal carcinoma has been shown to respond well to immune checkpoint inhibitors as a component of multiline treatment, with an objective response rate (ORR) of 10–33.3% (4–6). Additionally, targeted therapies are attracting interest owing to the comprehensive genomic characterization of ESCC. However, effective therapeutic targets are lacking in ESCC.

Traditionally, cancer cell lines and xenografts established from those cell lines have been widely used to evaluate new anticancer agents. This type of xenograft allows rapid analysis of the response of a tumor cell line to chemotherapy or other newly developed drugs. However, tumor cell lines have been cultured and passaged for many years, and the derived tumor xenografts may not truly represent a real tumor. Patient-derived xenograft (PDX) models, which are established by transplanting tumors from cancer patients directly into immunocompromised mice, have been used to evaluate the efficacy of targeted treatments for different types of tumors, such as those of breast cancer and non-small cell lung cancer (NSCLC) (7, 8). The models may more accurately represent a tumor in a real patient because they can retain the genetic information, immunohistological markers, and chemosensitivity of the primary tumor through a few passages. Therefore, PDX models have an advantage over cell line-based xenograft models (9–12). Indeed, these models are becoming the preferred preclinical tool used to study specific anticancer therapies for cancer patients as well as biological and genetic alterations (13).

Angiogenesis has increasingly been found to play a critical role in the development and progression of various cancers. Anlotinib, a small molecule multitarget tyrosine kinase inhibitor (TKI), suppresses tumor development and angiogenesis by directly inhibiting multiple targets, including PDGFR, VEGFR, c-Kit, and FGFR (14). A multicenter phase III study in 437 patients with advanced NSCLC (NCT02388919) (15) indicated that anlotinib can significantly prolong OS (9.6 vs. 6.3 months) and progression-free survival (PFS) (5.4 vs. 1.4 months) as compared with placebo. Another clinical study also indicated that anlotinib is an effective treatment for advanced soft tissue sarcoma (NCT01878448) (16). However, no reports have addressed anlotinib treatment in ESCC.

In this study, we successfully established PDX models of ESCC from tumor tissues to confirm the tolerability and efficacy of anlotinib in ESCC.

## Methods

### Patients and Tissue Samples

This study was approved by the research ethics board of our institute, and written informed consent for the use of the tissue samples and the publication of any potentially identifiable images or data was obtained from every patient before enrollment in the study. Between August 2018 and January 2019, 15 patients were enrolled in this study. All patients in this study had pathologically proven ESCC and available tissue, and none had been scheduled to undergo radiotherapy or chemotherapy before surgery. Tumor tissue was obtained intraoperatively from the edge of the whole tumor mass and transported to the animal laboratory in transport medium [fetal bovine serum (FBS)-free Roswell Park Memorial Institute (RPMI)-1640 medium supplemented with penicillin and streptomycin] under sterile conditions.

### Establishment of the Patient-Derived Xenograft Models

The tumor samples were rinsed in a Petri dish containing FBS supplemented with penicillin and streptomycin and carefully cut into small fragments (~30 mm^3^). Then, the fragments were subcutaneously implanted into five male NOD/SCID mice (5 weeks old) obtained from Beijing Vital River Laboratory Animal Technology Co., Ltd. Additional tumor tissue was cryopreserved [90% FBS and 10% dimethyl sulfoxide (DMSO)] and snap frozen in liquid nitrogen for future use. All procedures were performed in a certified biosafety hood and carried out in complete accordance with protocols approved by the Committee for the Care and Use of Laboratory Animals of our institute. Xenografts were assessed by palpation and Vernier calipers at least twice weekly for up to 6 months. The successfully established PDX model was named passage 1 (P1). Mice were kept until the tumor volume reached 700–800 mm^3^ if the implanted tumors grew. At that point, mice were decapitated, and the tumor samples were re-implanted into other mice following the same protocol as previously described. Subsequent passages were sequentially named P2, P3, and P4. The remaining tissues were stored at −80°C for subsequent detection and future usage.

### Treatment

When the tumors reached 100 mm^3^, 75 mice bearing P4 xenografts from three patients were randomly divided into five groups, as follows: a control group receiving normal saline (*n* = 15), a group receiving radiotherapy (dose: 5 Gy × 4, *n* = 15), a group receiving cisplatin combined with radiotherapy (5 Gy × 4, *n* = 15), a group receiving anlotinib combined with radiotherapy (5 Gy × 4, *n* = 15), and a group receiving anlotinib and cisplatin combined with radiotherapy (5 Gy × 4, *n* = 15). Mice in the treatment groups were anesthetized and subjected to local irradiation (5 Gy) to the tumors once daily for a total of four times. Cisplatin (2 mg/kg) was administered intraperitoneally once weekly, whereas anlotinib (2 mg/kg) was administered orally once daily for 2 weeks. Tumor volumes and body weights were measured three times weekly. The tumor volumes were calculated using the formula *V* = LD × (SD)2/2, where *LD* is the longest tumor diameter and *SD* is the shortest tumor diameter.

### H&E Staining and Immunohistochemistry

For histopathological assessment, primary tumors and xenografts were embedded in paraffin blocks and then stained with hematoxylin and eosin (H&E). All tissue sections were stained with an H&E staining kit (C0105, Beyotime, China) after the 5-μm sections were deparaffinized with dimethylbenzene according to a standard method and were evaluated by two independent pathologists.

For immunohistochemistry (IHC), tissue sections were deparaffinized and hydrated. After antigen retrieval with sodium citrate antigen retrieval solution (pH = 6.0) and blocking with 3% bovine serum albumin (BSA), sections were hybridized with a primary antibody (specific for c-PARP, BAX, PCNA, or CD31) overnight at 4°C. Then, a horseradish peroxidase (HRP)-conjugated secondary antibody (recognizing the appropriate primary antibody species) was added and incubated at room temperature for 50 min. Tissue sections were developed with freshly prepared 3,3′-diaminobenzidine (DAB) chromogenic reagent and counterstained with hematoxylin staining solution for 3 min. Finally, sections were dehydrated successively in a graded series of 75, 85, and 100% ethanol and mounted with resin mounting medium. Nuclei stained with hematoxylin appear blue, and positive cells developed with DAB reagent appear brownish yellow. The results were obtained based on the average of any four fields in 200 times. All sections were observed by microscopy and analyzed using the Image-Pro Plus 6.0 software program (Media Cybernetics, Rockville, MD, USA).

### TUNEL Assay

Tissue sections were deparaffinized and rehydrated. After antigen retrieval with proteinase K working solution and permeabilization with permeabilization working solution, a mixture of TdT and dUTP at a ratio of 1:9 was added to the slides and incubated at 37°C for 2 h for the TUNEL reaction. Then, the endogenous peroxidase activity was blocked, and the tissues were covered with reagent 3 (converter-POD). Freshly prepared DAB chromogenic reagent was added to the tissue sections, which were then counterstained with hematoxylin staining solution for 3 min. Finally, the sections were dehydrated successively in a gradient of 70, 80, 95, and 100% ethanol followed by xylene and were mounted with resin mounting medium. Nuclei stained with hematoxylin appear blue, and positive cells developed with DAB reagent appear brownish yellow. All sections were observed by microscopy and analyzed using the Image-Pro Plus 6.0 software program (Media Cybernetics, Rockville, MD, USA).

### Protein Chip Technology

Plasma was isolated from blood that was sampled from mice eyes before these mice were euthanized at the end of the 2-weeks treatment and stocked at −80°C. Quantibody® Mouse Cytokine Antibody Array 4000 (QAM-CAA-4000), a combination of five non-overlapping arrays to quantitatively measure 200 mouse cytokines, was employed for automatically detect cytokines from 200 μl of mouse plasma by Guangzhou RayBiotech Biotechnology Co., Ltd, in 1 week. A reagent kit of GSM-CAA-4000 was first used in our study. Briefly, the glass slides were air dried completely. After blocking and incubation, the reconstituted detection antibody was added to 1.4 ml of sample diluent and centrifuged briefly. Then, 80 μl of the mixture was added to each well and incubated at room temperature for 1–2 h. After a brief centrifugation, 1.4 ml of sample diluent was added to a tube containing Cy3 equivalent dye-conjugated streptavidin. Then, 80 μl of the Cy3 equivalent dye-conjugated streptavidin mixture was added to each well and incubated in a dark room at room temperature for 1 h. Finally, the signals were visualized in an InnoScan 300 Microarray Scanner equipped with a Cy3 filter (green channel). After the operation was done, GSM-CAA-4000 data analysis software was used for data analysis. Primarily, the original data were normalized with software, and then normalization data were selected for analysis. The basic statistics used for significance analysis was moderated *t*-statistic. Analysis results included (log2) fold changes, *P*-values, and adjusted *P*-values for each protein and for each contrast individually. Heatmap was conducted on all differential expression proteins between two groups. Euclidean distance and complete cluster were used to analyze dissimilarities between two groups, and then the first two principal components were plotted to show the difference between two groups. In this way, cytokines were selected automatically.

### Patients

Two patients with ESCC were enrolled in our study. With full informed consent, patients undergoing radiotherapy received at least 50 Gy of radiation and concurrent chemotherapy with either carboplatin and paclitaxel, or cisplatin and 5-fluorouracil plus anlotinib [12 mg, D1–14, every 3 weeks (Q3W)]. We retrospectively analyzed the tolerability and efficacy of anlotinib combined with chemoradiotherapy in these two patients.

### Statistical Analysis

Data are presented as the means ± standard errors of the mean (SEMs). Statistical analyses were performed using Student's *t*-test to compare the differences between two groups in SPSS 22.0 software (IBM, USA). *P* < 0.05 was considered statistically significant.

## Results

### Patient Characteristics

A total of 15 samples of ESCC (two in the upper segment, nine in the middle segment, and four in the distal segment) were obtained by surgical resection. Ultimately, three PDX models were successfully established in NOD/SCID mice. Table 1 lists the baseline characteristics of the subjects. The donor patients included 12 men (80%) and three women (20%) with a median age of 61 years (range, 46–74). Seven patients had lymph node (LN) metastasis, and no patient had been treated before surgery. According to the American Joint Committee on Cancer (AJCC) 8th staging system, eight (45%) patients were diagnosed with stage II disease, and seven patients (65%) were diagnosed with stage III/IV disease. Among these patients, four samples (27%) were well-differentiated, eight samples (53%) were moderately differentiated, and three samples (20%) were poorly differentiated.

**Table 1:** Clinical characteristics of studied patients.

**No**.	**Gender**	**Age** **(years)**	**Tumor** **differentiation**	**TNM** **staging**	**Tumor** **location**	**Engrafted** **mode**	**Written informed** **consent**
EG1	Male	69	Moderate	T3N0M0 IIB	Distal	No	Individual
EG2	Male	67	Well	T3N1M0 IIIB	Middle	No	Individual
EG3	Male	69	Well	T4N2M0 IVA	Distal	Yes	Individual
EG4	Male	74	Moderate	T2N0M0 IIA	Distal	No	Individual
EG5	Female	58	Moderate	T3N1M0 IIIB	Middle	No	Individual
EG6	Male	58	Poor	T3N2M0 IIIB	Distal	Yes	Individual
EG7	Male	71	Poor	T3N0M0 IIB	Middle	No	Individual
EG8	Male	46	Moderate	T3N1M0 IIIB	Middle	Yes	Individual
EG9	Male	67	Moderate	T3N0M0 IIB	Middle	No	Individual
EG10	Female	62	Moderate	T3N0M0 IIB	Middle	No	Individual
EG11	Male	56	Well	T2N2M0 IIIB	Middle	No	Individual
EG12	Male	56	Poor	T3N0M0 IIA	Middle	No	Individual
EG13	Female	58	Well	T3N0M0 IIA	Upper	No	Individual
EG14	Male	58	Moderate	T3N1M0 IIIA	Upper	No	Individual
EG15	Male	61	Moderate	T3N0M0 IIB	Middle	No	Individual

### Establishment of Patient-Derived Xenograft Models

Three transplanted xenografts were successfully passaged to the fourth generation (range, 6.1–9.5 months), and the overall transplantation rate for PDXs was 20% (3/15) in our study. The transplantation rate was increased along with serial passaging: 27% (4/15), 75% (3/4), 100% (3/3), and 100% (3/3) for P1, P2, P3, and P4 PDXs, respectively. Additionally, the median latency period for the third passage was shorter than the first and second passage PDXs: 76.52 ± 18.17, 54.96 ± 16.24, and 29.15 ± 15.96 days for P1, P2, and P3, respectively. After the fourth generation, the PDX models exhibited robust growth and stability without further changes in model formation; thus, they could be used in future studies.

### The Histology Was Consistent Between the Original Patient Tumors and the Xenografts

**Figure 1:**
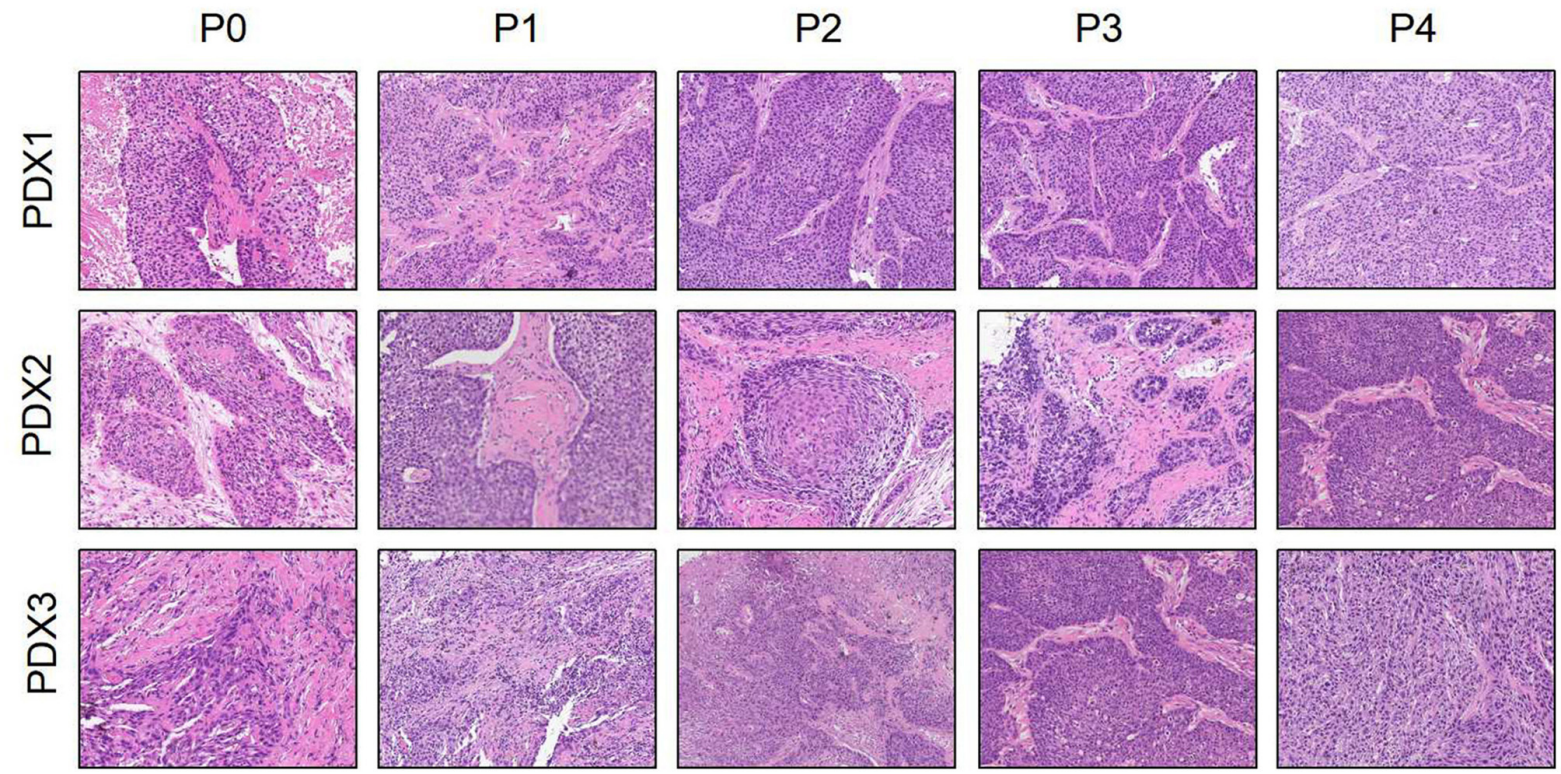
Histological section (H&E) presented that the esophageal patient tissue (P0) was consistent with the series passages (P1, P2, P3, and P4) in the SCID mice (×200).

**Figure 2:**
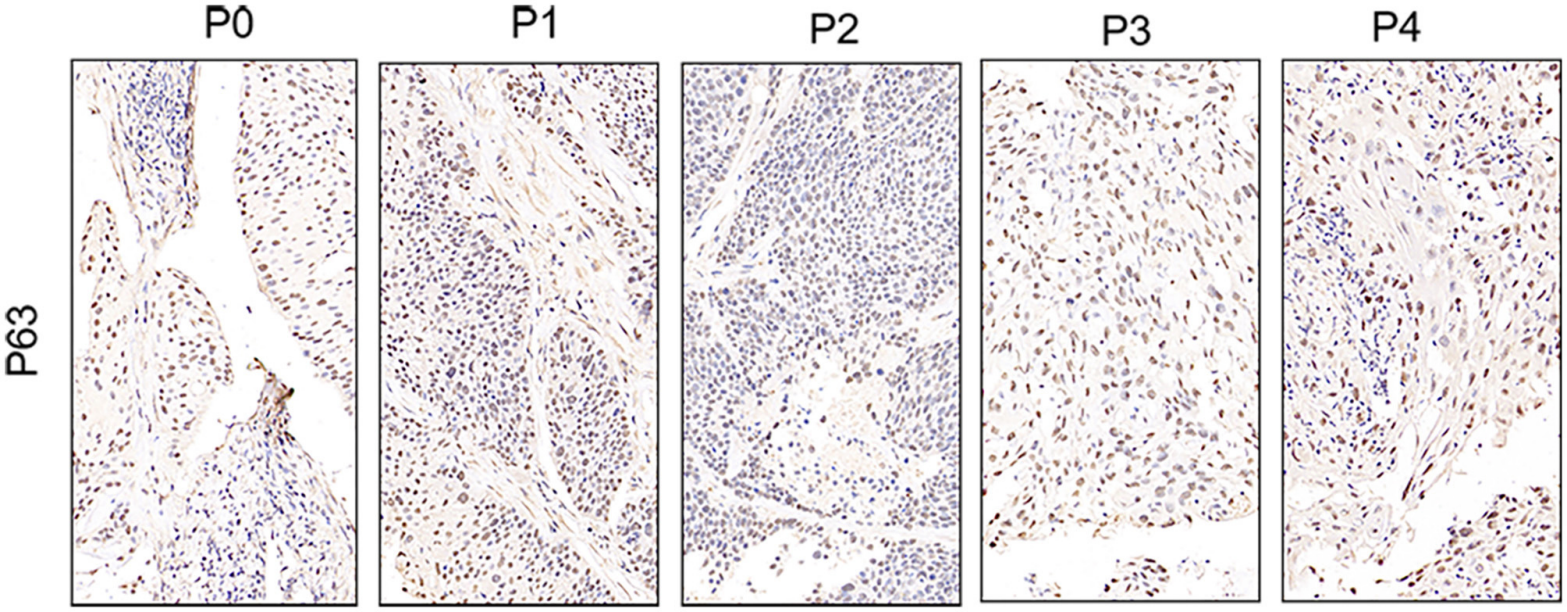
Immunohistochemistry presented that P63 was expressed in the esophageal patient tissue (P0) and all the series passages (P1, P2, P3, and P4). The figure provided is a representative image, and other images are provided in the Supplementary Files; ×400.

To further assess the PDX xenografts, we initially compared the histology of the original patient tumors with that of the corresponding serially passaged xenografts by H&E staining. The pathological characteristics of the passaged xenografts were consistent with those of the original patient tumor tissues (Figure 1). In addition, the P63 gene and its encoded protein play important roles in the early physiological and pathological course of disease in the esophageal mucosa and may be helpful in the early diagnosis and prognostic evaluation of ESCC. Our immunohistochemical staining results proved that the expression of P63 was positive in all P4 PDXs, further proving the squamous phenotype of the tumors (Figure 2).

### Efficacy of Different Types of Treatment Regimens in Patient-Derived Xenograft Models

**Figure 3:**
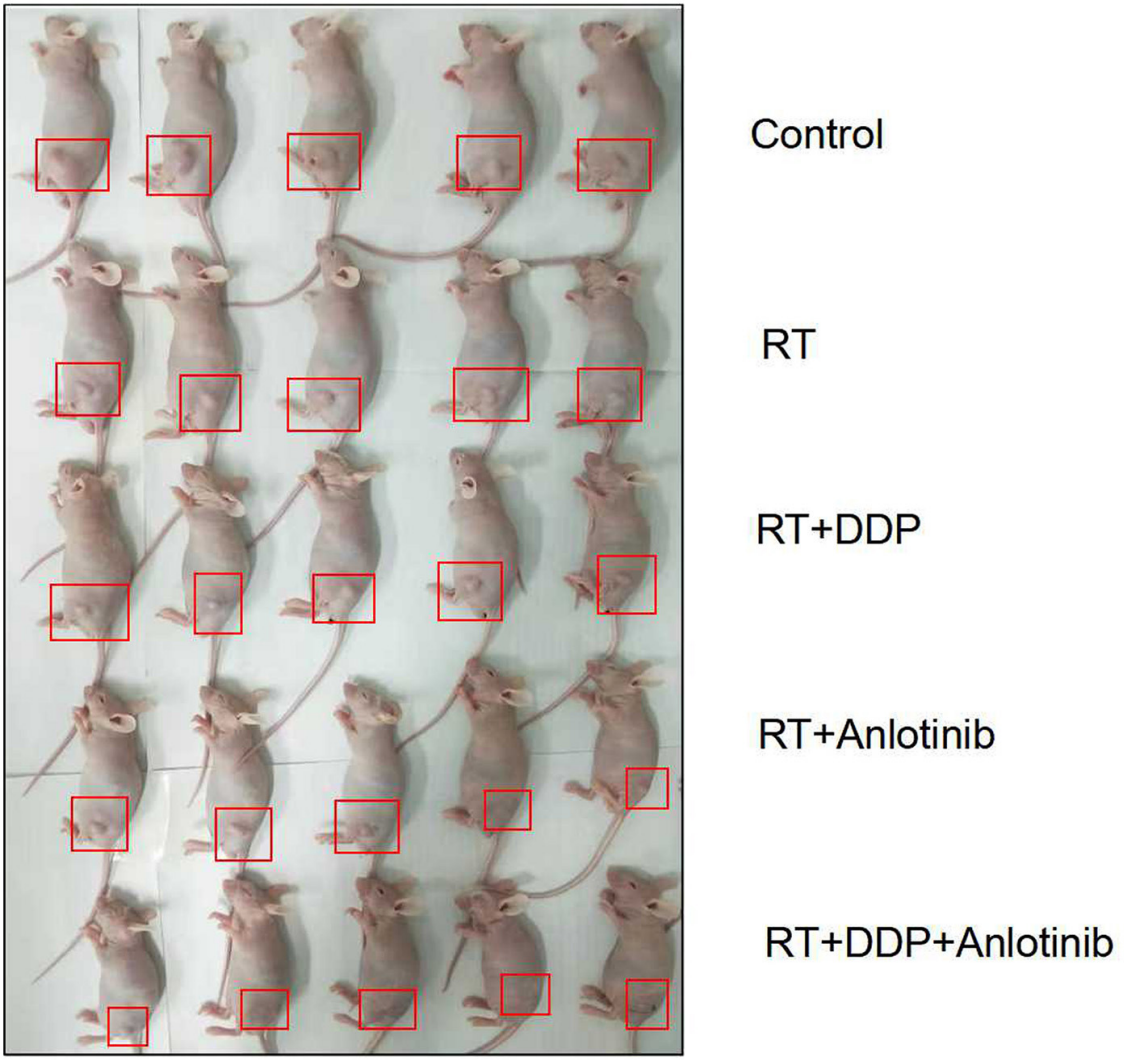
Intuitive antitumor efficacy in patient-derived xenograft (PDX) models showed that the tumor in the anlotinib combined with chemoradiation-treated group shrank the most.

Three PDX models were used in our study, and the mice in every model (25 mice per model) were randomly divided into five groups. The detailed data of tumor volumes and body weights are listed in Table 2. The tumor growth inhibitory rate compared with that in the control group receiving normal saline (*n* = 15) decreased in the following order: anlotinib and cisplatin combined with radiotherapy (5 Gy × 4, *n* = 15) group > anlotinib combined with radiotherapy (5 Gy × 4, *n* = 15) group > cisplatin combined with radiotherapy (5 Gy × 4, *n* = 15) group ≥ radiotherapy only (5 Gy × 4, *n* = 15) group. Our results showed that the differences in the tumor volumes between the control group and each of the four treatment groups were significant (all *P* = 0.000). The weight was not significantly different between the control group and the other treatment groups (*P* > 0.05). The picture of intuitive antitumor efficacy in PDX models showed that the xenografts in chemoradiotherapy combined with anlotinib group shrank the most compared than did control and the other three treatment groups (Figure 3).

**Table 2:** The detailed data of tumor volume and body weight.

**No**.	**Tumor volume**	**Control group**	**RT**	**RT + DDP**	**RT + Anlotinib**	**RT + DDP + Anlotinib**
	Initial	266.64 ± 37.05	243.49 ± 31.38	252.61 ± 60.13	260.90 ± 43.67	267.24 ± 51.13
PDX1	Final	343.51 ± 29.22	188.54 ± 40.04	150.30 ± 15.87	144.29 ± 15.05	121.02 ± 25.72
	Changing	77.06 ± 32.90	−54.95 ± 39.63	−102.31 ± 46.44	−116.61 ± 45.17	−146.23 ± 48.59
	Initial	187.52 ± 62.04	171.50 ± 31.30	185.45 ± 44.30	178.06 ± 40.02	181.31 ± 15.67
PDX2	Final	246.07 ± 109.69	116.38 ± 25.17	133.11 ± 53.32	119.17 ± 30.60	71.79 ± 5.88
	Changing	58.55 ± 64.42	−55.11 ± 22.68	−52.34 ± 55.52	−58.89 ± 38.21	−109.52 ± 18.05
	Initial	177.32 ± 52.96	160.93 ± 27.51	169.28 ± 42.44	144.01 ± 86.47	162.29 ± 22.77
PDX3	Final	261.34 ± 116.80	122.33 ± 54.90	130.60 ± 55.06	111.17 ± 26.33	77.78 ± 17.65
	Changing	84.01 ± 72.90	−38.60 ± 35.07	−38.68 ± 55.06	−32.90 ± 73.32	−84.51 ± 20.69
**NO**.	**Weight**	**Control group**	**RT**	**RT** **+** **DDP**	**RT** **+** **Anlotinib**	**RT** **+** **DDP** **+** **Anlotinib**
	Initial	25.92 ± 22.15	24.85 ± 2.06	26.3 ± 41.20	24.7 ± 81.34	24.29 ± 1.57
PDX1	Final	25.99 ± 0.50	24.77 ± 2.30	25.9 ± 62.02	23.9 ± 12.47	23.45 ± 2.27
	Changing	0.06 ± 2.53	−0.08 ± 0.73	−0.88 ± 1.84	−0.87 ± 2.56	−0.83 ± 1.39
	Initial	22.68 ± 1.79	22.25 ± 0.84	22.66 ± 1.08	22.32 ± 1.48	22.71 ± 2.71
PDX2	Final	24.07 ± 0.77	23.35 ± 1.82	23.03 ± 1.01	22.05 ± 1.87	23.29 ± 1.47
	Changing	1.60 ± 1.23	1.10 ± 1.21	0.37 ± 0.84	−0.27 ± 1.17	0.57 ± 1.46
	Initial	22.00 ± 2.54	21.23 ± 2.77	20.86 ± 2.92	21.99 ± 2.70	22.05 ± 2.58
PDX3	Final	24.21 ± 1.31	22.10 ± 1.30	22.86 ± 0.97	22.84 ± 1.07	22.69 ± 0.95
	Changing	2.21 ± 2.33	1.16 ± 1.72	2.00 ± 3.17	0.85 ± 3.15	0.64 ± 3.11

*RT, radiation therapy; DDP, cisplatin*.

We even found that some tumors have shrunk so much that it was hard to distinguish them in chemoradiotherapy combined with anlotinib group.

**Figure 4:**
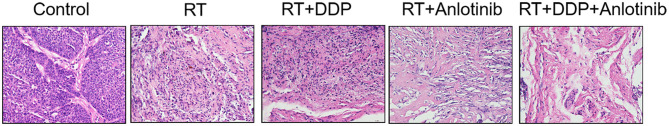
Representative images during treatment showed that the structure of tumor tissue in control group remained normal with tumor and mesenchymal cells. The structure of other treatment groups was showed to be destroyed with various degrees in which anlotinib combined with chemoradiotherapy group exhibited the most significant change. The figure provided is a representative image, and other images are provided in the Supplementary Files. RT, radiation therapy; DDP, cisplatin.

**Figure 5:**
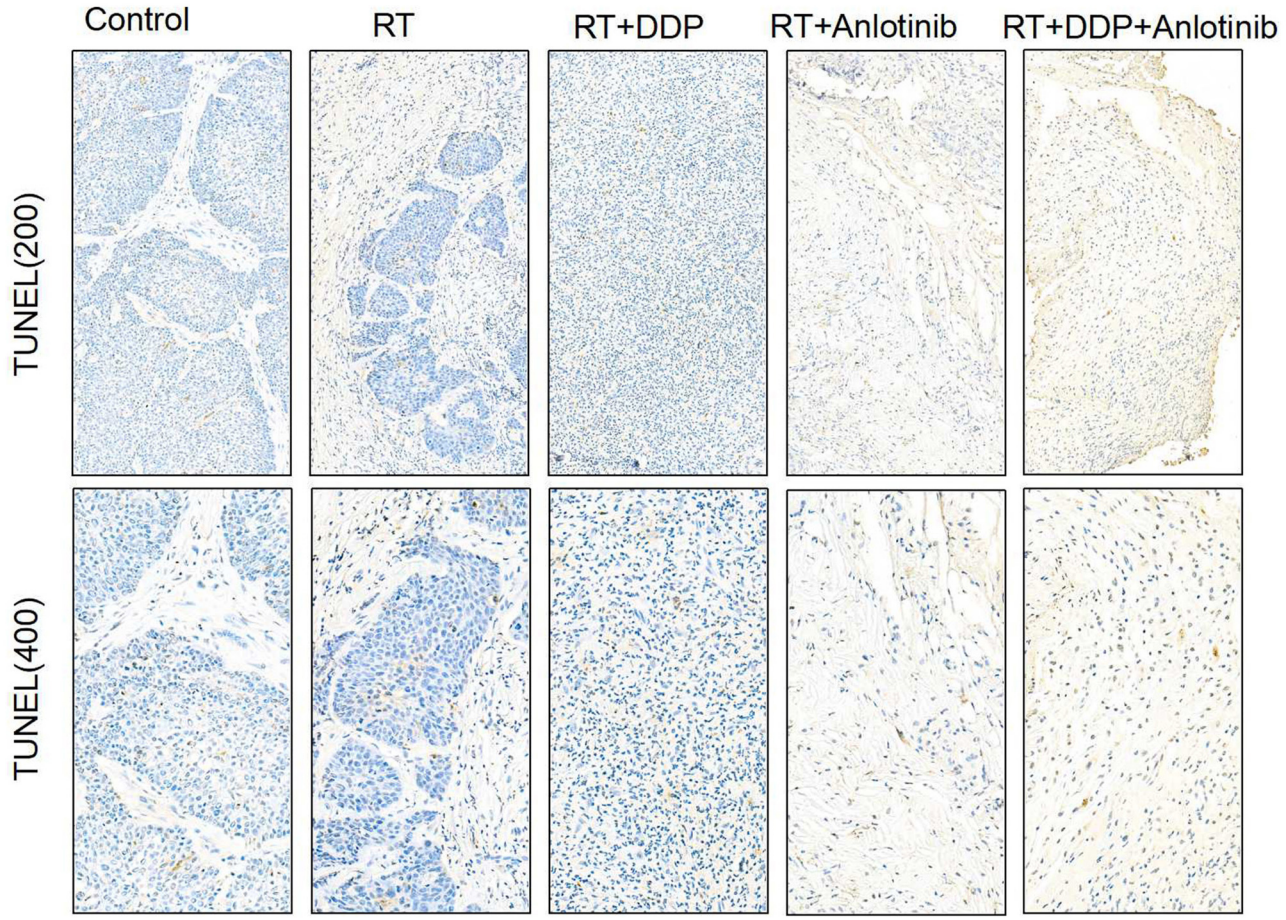
Representative images of TUNEL assay showed that brownish yellow nuclei indicate the apoptotic cells. The four treatment groups showed different apoptosis rates in which anlotinib combined with chemoradiation-treated group was the highest compared with the control group. The figure provided is a representative image, and other images are provided in the Supplementary Files. RT, radiation therapy; DDP, cisplatin.

**Figure 6:**
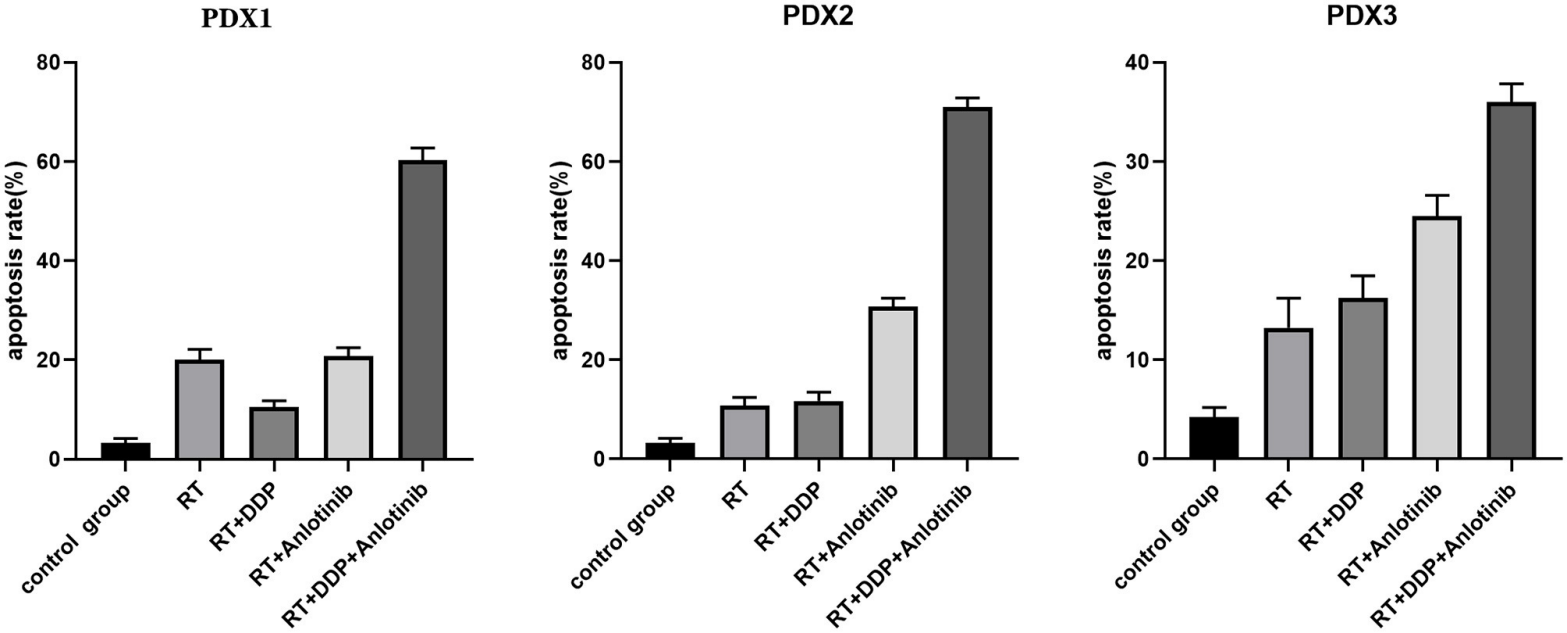
The quantified apoptosis rates of different groups in patient-derived xenograft (PDX) models. The data are shown as mean ± *SD*. Compared with the control group, apoptosis rates at different degrees existed in four treatment groups, and anlotinib combined with chemoradiation-treated group had the highest apoptosis rate. RT, radiation therapy; DDP, cisplatin.

**Figure 7:**
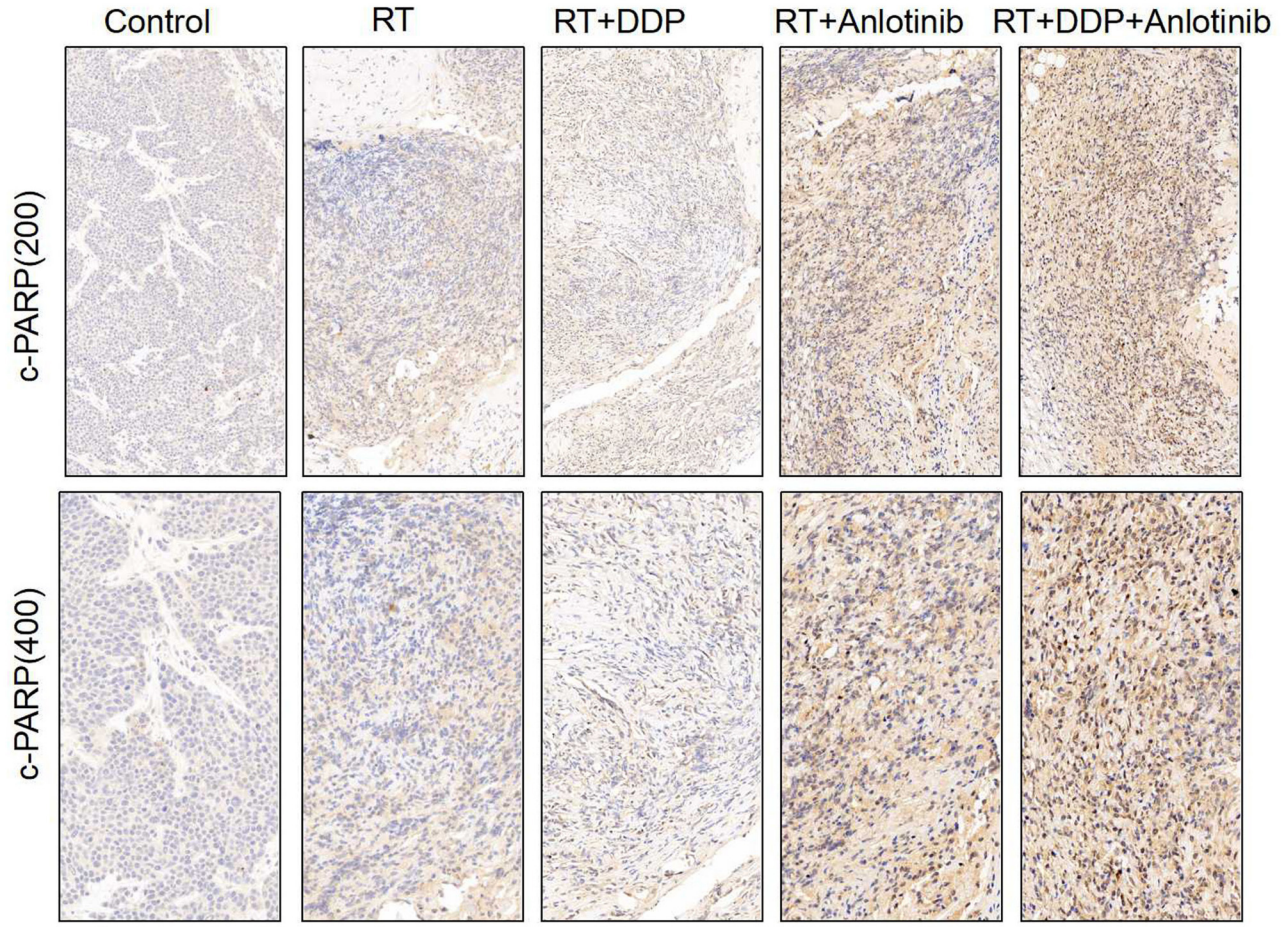
Representative images for c-PARP immunohistochemistry show that brownish yellow nuclei and cytoplasm staining, respectively, indicate the positive cells. Varying degrees of positive rates were shown for the four treatment groups in which anlotinib combined with chemoradiation-treated group was the highest compared with the control group. The figure provided is a representative image, and other images are provided in the Supplementary Files. RT, radiation therapy; DDP, cisplatin.

**Figure 8:**
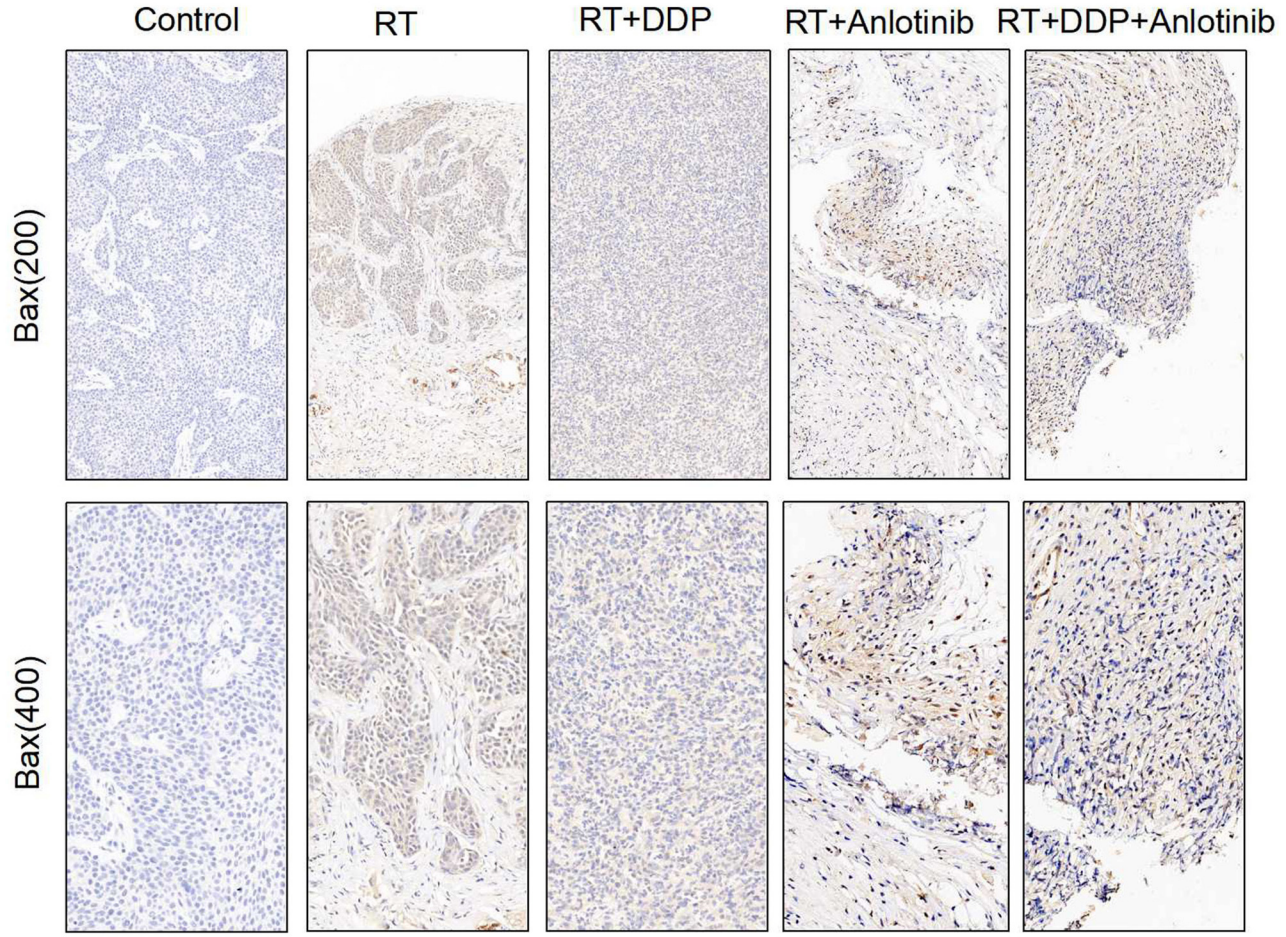
Representative images for Bax immunohistochemistry show that brownish yellow nuclei and cytoplasm staining, respectively, indicate the positive cells. Varying degrees of positive rates were shown for the four treatment groups in which anlotinib combined with chemoradiation-treated group was the highest compared with the control group. The figure provided is a representative image, and other images are provided in the Supplementary Files. RT, radiation therapy; DDP, cisplatin.

**Figure 9:**
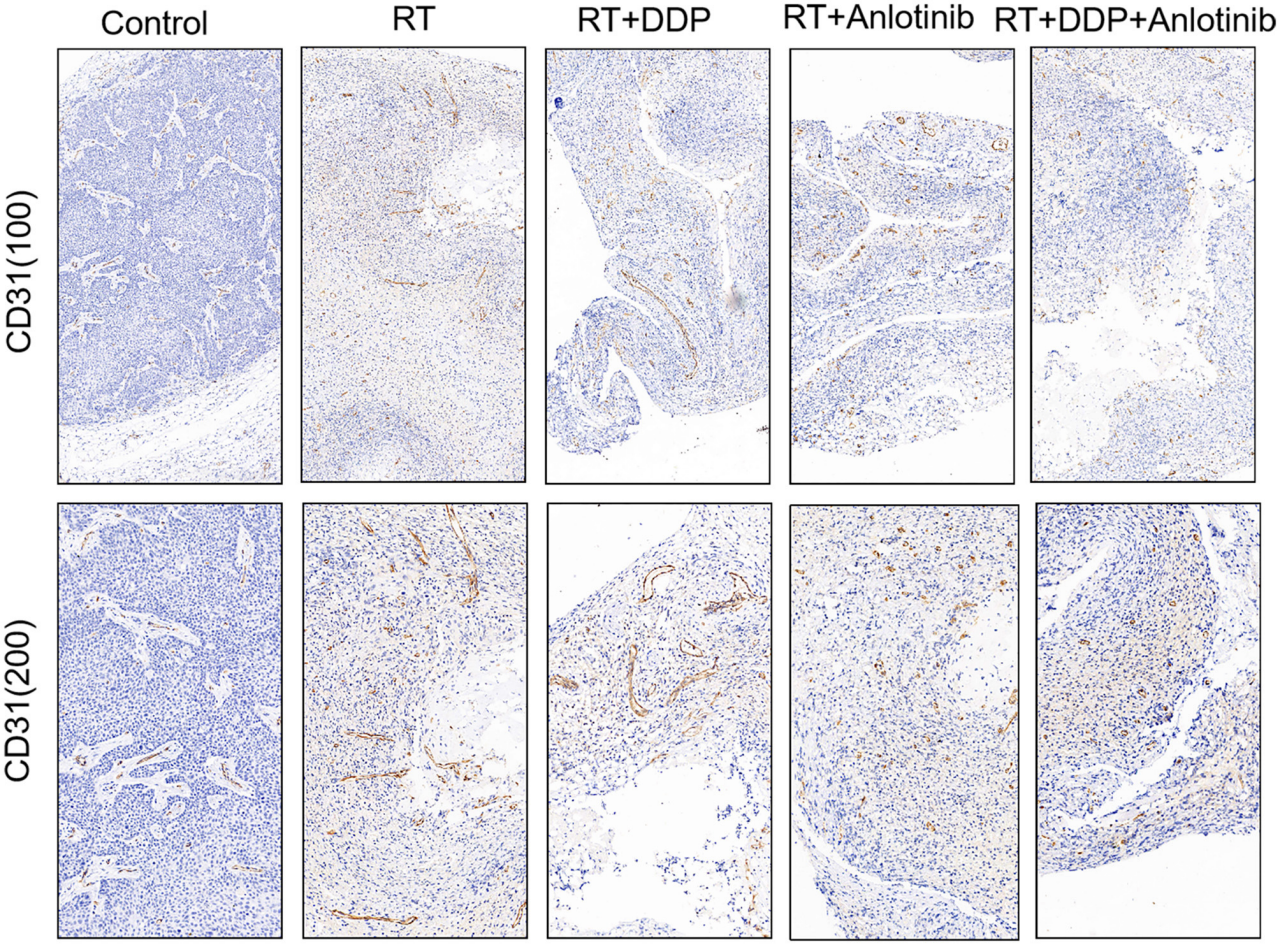
Representative images of changes in the number of vessels showed that the four treatment groups showed different numbers of vessels in which anlotinib combined with chemoradiation-treated group was the lowest compared with the control group. The figure provided is a representative image, and other images are provided in the Supplementary Files. RT, radiation therapy; DDP, cisplatin.

**Figure 10:**
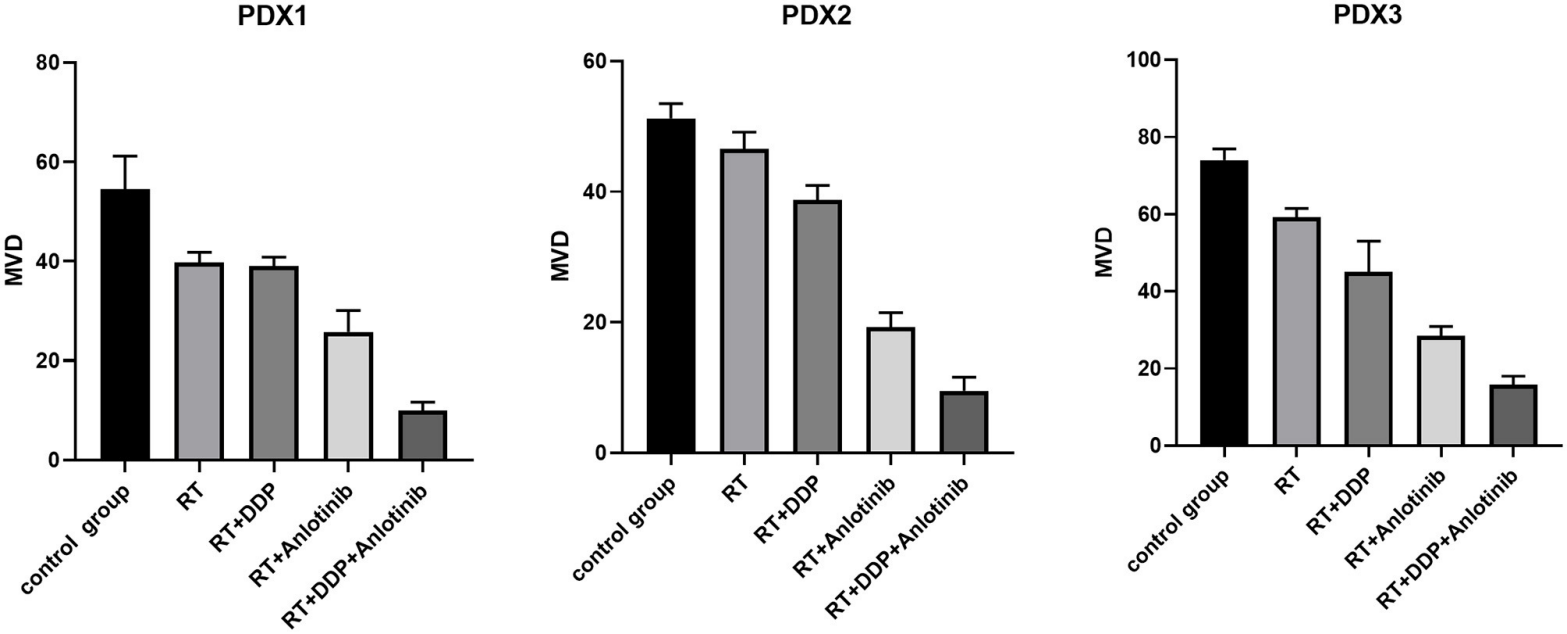
The microvessel density (MVD) of different groups in patient-derived xenograft (PDX) models. The number of vessels were quantified by MVD, and the data are shown as mean ± *SD*. Compared with the control group, differences of MVD at various degrees existed in four treatment groups, and anlotinib combined with chemoradiation-treated group had the lowest MVD. RT, radiation therapy; DDP, cisplatin.

**Figure 11:**
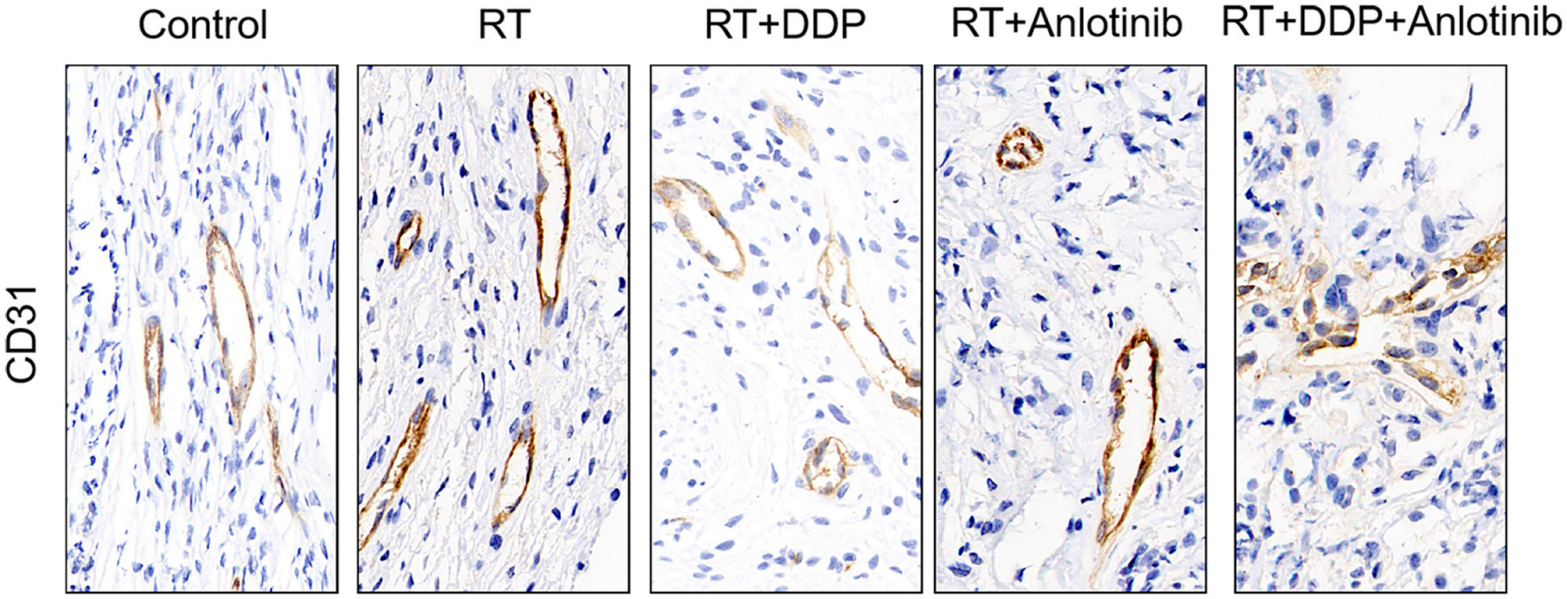
Representative images during treatment showed that the vascular structures in the control group retained their normal shape. However, the vessels showed pucker, and the vascular structures were damaged to different extent in the four treatment groups where the anlotinib combined with chemoradiation-treated group exhibited the most significant change. The figure provided is a representative image, and other images are provided in the Supplementary Files. RT, radiation therapy; DDP, cisplatin.

**Figure 12:**
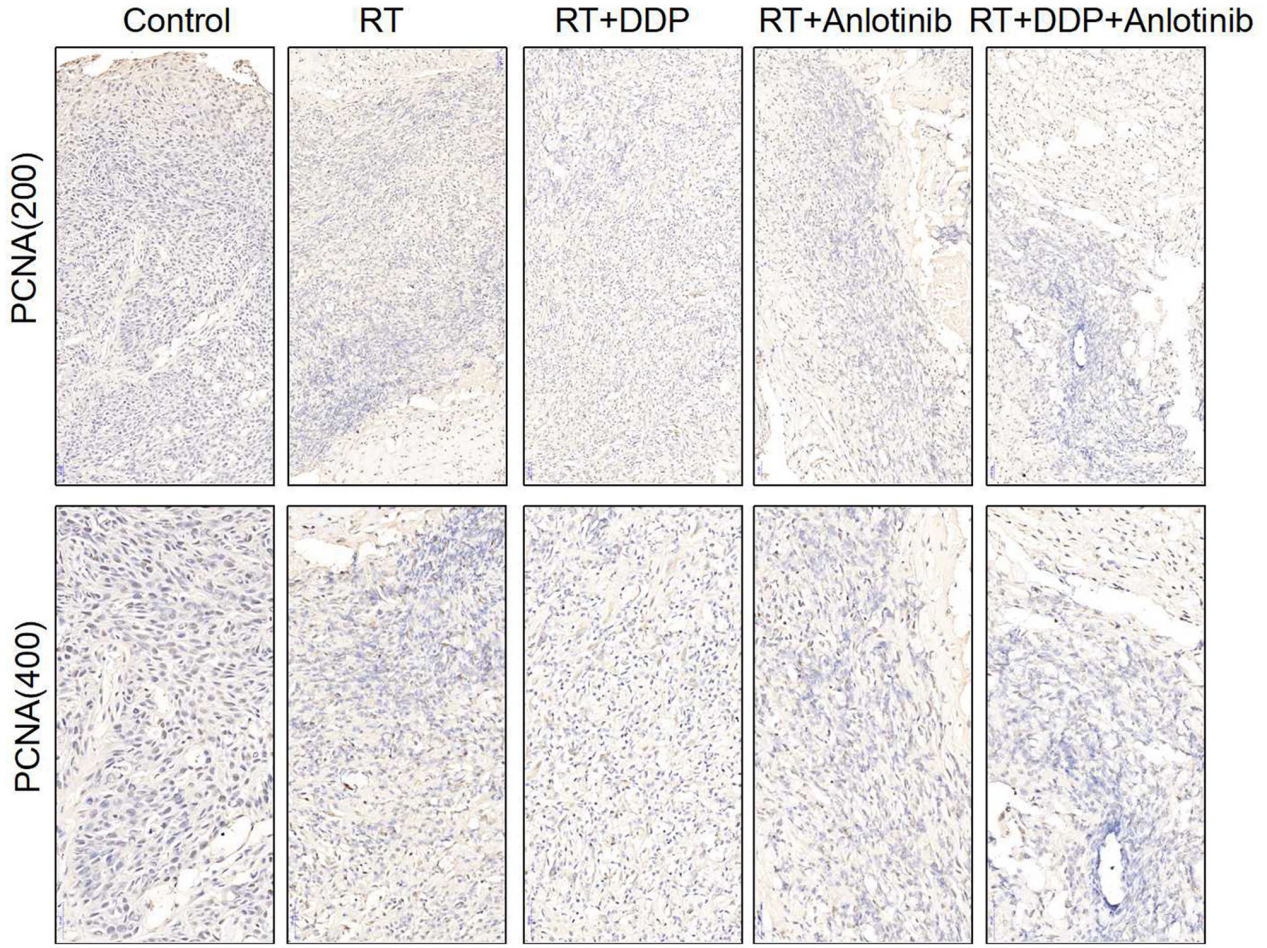
Representative images for proliferation marker of PCNA show that brownish yellow nuclei indicate the positive cells. Various degrees of positive rates were shown in the four treatment groups in which anlotinib combined with chemoradiation-treated group was the lowest compared with control group. The figure provided is a representative image, and other images are provided in the Supplementary Files. RT, radiation therapy; DDP, cisplatin.

The group treated with anlotinib combined with chemoradiotherapy showed more cancer cell apoptosis and antiangiogenic effects in the tumor microenvironment than the other groups. H&E staining, TUNEL assay, and IHC were performed within first a week after treatment. H&E staining showed that the structure and shape of tumor cells clearly exhibited the most significant change, with necrotic cells, in the group treated with anlotinib combined with chemoradiotherapy compared with the other treatment groups as well as with the control group (Figure 4). Then, we evaluated apoptosis via both TUNEL and apoptotic marker (c-PARP and Bax) immunoreactivity in various groups. The apoptosis rate in the anlotinib combined with chemoradiation-treated group was the highest and was significantly higher than that in the control group and the other treatment groups (radiotherapy only, cisplatin combined with radiotherapy, and anlotinib combined with radiotherapy groups, sequentially) (Figures 5, 6). The immunohistochemical staining results showed that the c-PARP and Bax levels were increased in the treatment groups compared with the control group, and the anlotinib combined with chemoradiation-treated group exhibited the highest levels (Figures 7, 8). Furthermore, anlotinib can exert antitumor effects via its antiangiogenic activity, and CD31 is a tumor endothelial marker. Compared with that in the tumor masses treated with normal saline and other treatments, the decrease of CD31-immunolabeled cells in the tumor masses treated with anlotinib combined with chemoradiotherapy and anlotinib combined with radiotherapy was significant (*P* < 0.05) (Figures 9, 10). And results showed that more abnormalities architecture of carcinoma vascularities existed in anlotinib combined with chemoradiotherapy and anlotinib combined with radiotherapy groups compared with those in control group and other two treatment groups (Figure 11). In addition, cells immunoreactive for the proliferation marker PCNA were significantly decreased in the treated tumor masses compared with the normal saline-treated tumor masses, and anlotinib combined with chemoradiation treatment resulted in the lowest PCNA levels (Figure 12). These results suggested that anlotinib combined with chemoradiation exhibits a significant antitumor activity by promoting the apoptosis and inhibiting the angiogenesis of ESCC cells.

### Anlotinib Induced a Much Stronger Response Than Other Treatments in Mice, as Reflected by the Release of Various Cytokines

**Figure 13:**
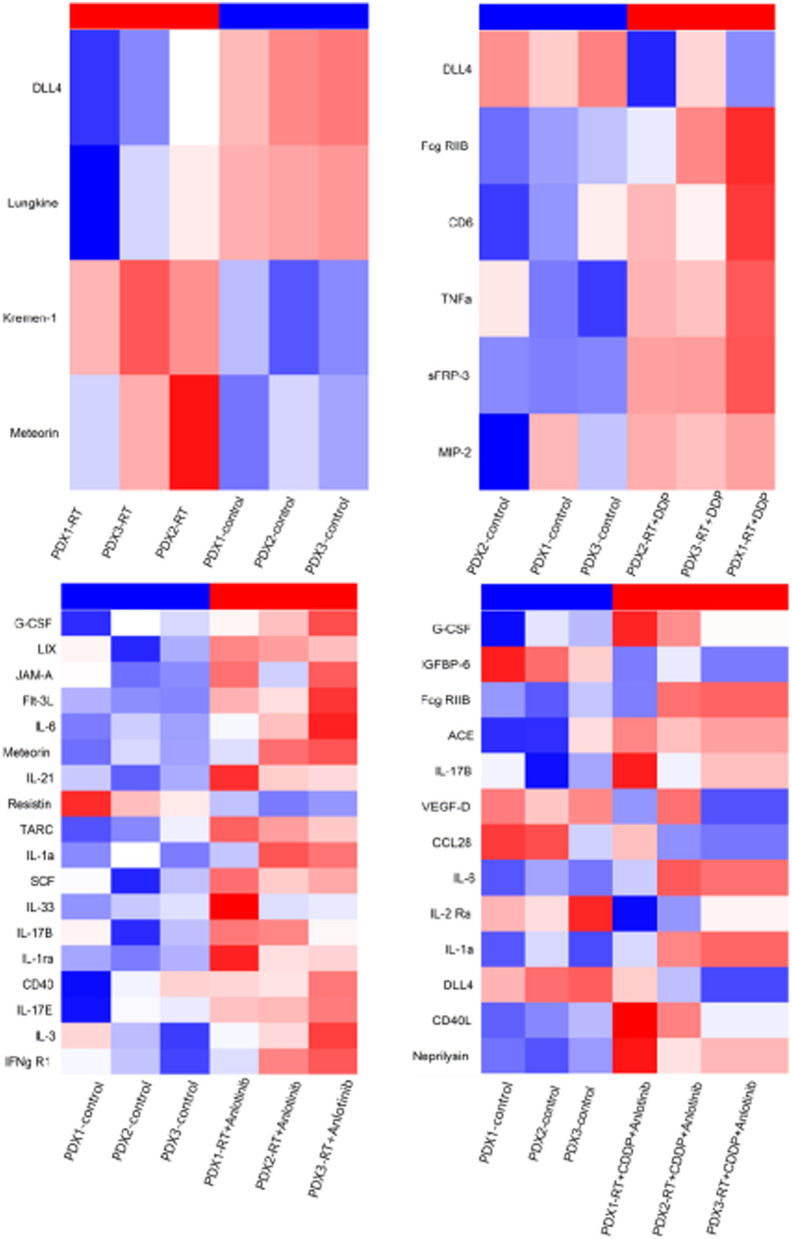
Heatmaps show the released cytokines in plasma from four treatment groups compared with control group. RT, radiation therapy; DDP, cisplatin.

Cytokines play a significant role in apoptosis, angiogenesis, innate immunity, and cell growth and differentiation. They are involved in interactions between different cell types, cellular responses to environmental conditions, and the maintenance of homeostasis. The body could respond to changes in the tumor by cytokines alteration in plasma. Plasma cytokine profiling may alter greater as the tumor shrank obviously. We took the control group as a benchmark and compared the other four treatment groups to the control group to evaluate the relative intensity of the body's response. As a result, these responses could indirectly verify the therapeutic efficacy of different groups to some extent in our study. Therefore, we conducted cytokine antibody array analyses of plasma and found that a much stronger response, with the release of more types of cytokines, was induced in the anlotinib-based treatment groups compared with the other groups (Figure 13). These results indirectly proved that ESCC showed a good response to anlotinib treatment.

### Anlotinib Combined With Chemoradiotherapy Showed Significant Antitumor Efficacy in Two Patients With Esophageal Squamous Cell Carcinoma

This study was approved by the research ethics board of our institute, and written informed consent was provided by two patients for the publication of any potentially identifiable images or data in this article before enrollment in the study. In addition, the clinical characteristics of these two patients are provided in Table 3.

**Table 3:** Clinical characteristics of two involved patients.

Characteristics	**Patient 1**	**Patient 2**
Gender	Male	Male
Age (year)	58	50
Tumor differentiation	Moderately	Moderately
TNM staging	ypT4N2M0	cT2N3M1
Tumor location	Upper	Middle
Anlotinib	12 mg, D1–14, Q3W	12 mg, D1–14, Q3W
Chemotherapy (two cycles)	Docetaxel (120 mg, D1, Q3W) Cisplatin (12 mg, D1–14, Q3W)	Docetaxel (120 mg D1 Q3W) Nedaplatin (60 mg D1–2 Q3W) Tegafur (50mg D1–14 Q3W)
Radiotherapy	60 Gy(30f)	50 Gy(25f)
Radiation field	Metastatic lymph nodes	Metastatic lymph nodes; Primary tumour
Site of metastatic lymph nodes	Retroperitoneu; Mediastinum	Mediastinum
Outcome	PR	PR

#### Case 1

**Figure 14:**
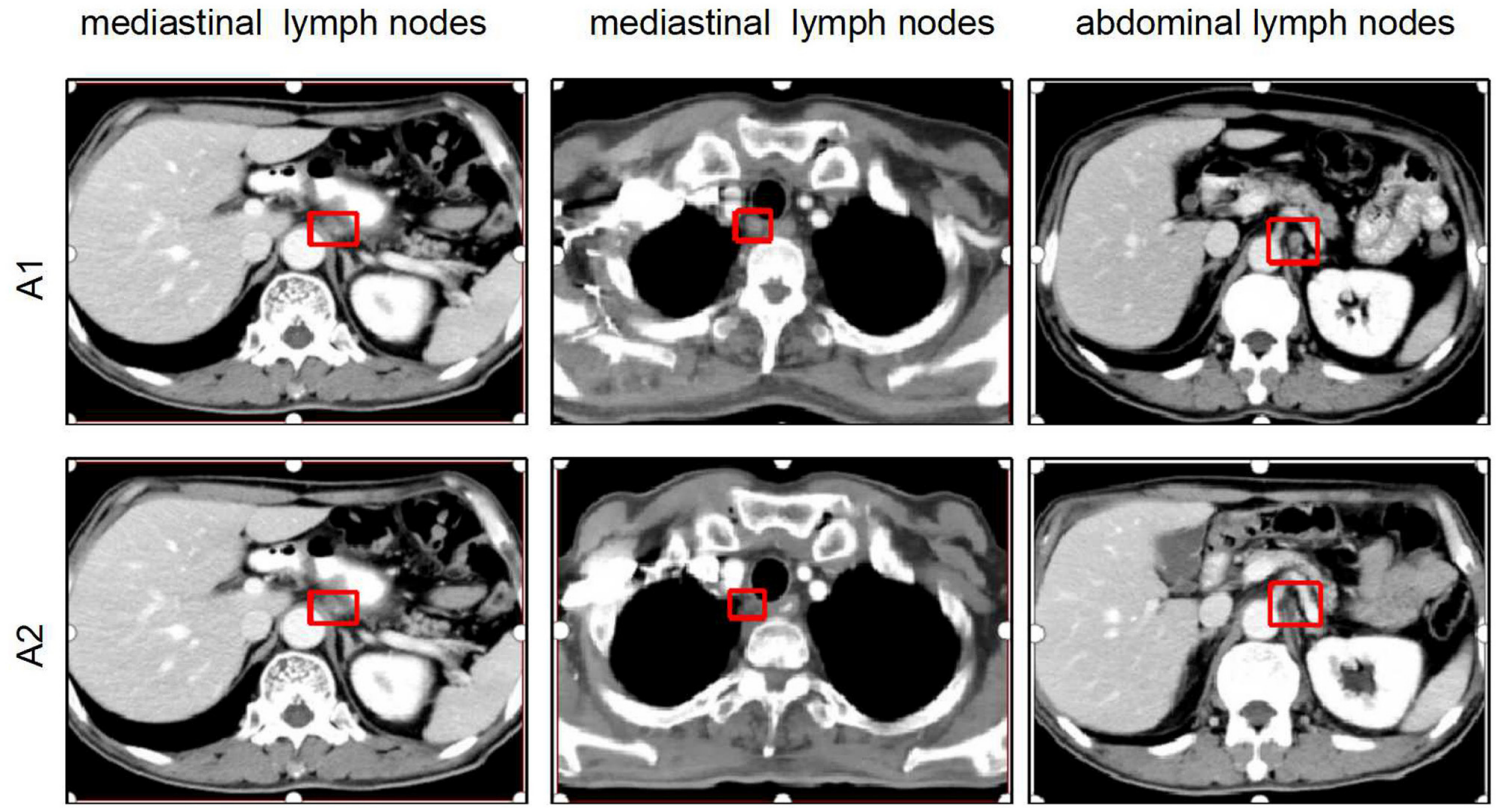
Comparison of chest CT scan between before and after treatment for the first patient. (A1) Chest CT scan showing enlarged lymph nodes in the retroperitoneum and mediastinum in postoperative re-examination. (A2) Chest CT showing that the enlarged lymph nodes in the retroperitoneum and mediastinum shrank or became loosened after 6 weeks of anlotinib combined chemoradiotherapy. CT, computed tomography; PD, progressive disease; PR, partial progressive disease.

A 58-years-old man diagnosed with ESCC received radical resection on July 31, 2018, after two cycles of first-line (800 mg tegafur, D1–5 in combination with 20 mg cisplatin, D1–5) and second-line neoadjuvant chemotherapy [120 mg of docetaxel in combination with 120 mg of cisplatin every 3 weeks (Q3W)]. The pathological diagnosis was moderately differentiated squamous carcinoma with a pathological stage of IIIB (ypT4N2M0). Chest computed tomography (CT) scanning of on postoperative September 3, 2018, showed a thickened esophageal wall in the left upper lobe and surgical anastomosis as well as multiple enlarged LNs in the supraclavicular region, retroperitoneum, and mediastinum (Figure 14). The patient was administered another two cycles of combination chemotherapy with docetaxel (120 mg, Q3W) and cisplatin (12 mg, D1–14, Q3W). Chest CT scan on October 19, 2018, indicated that the esophageal wall was thicker than at the last inspection and that the enlarged LNs had not significantly changed. The patient was given radiotherapy with 2-Gy fractions once daily for 5 days per week to a total dose of 60 Gy beginning on October 19, 2018. With full informed consent, the patient was administered capecitabine (1,250 mg/m^2^, Q3W) in combination with anlotinib (12 mg, D1–14, Q3W) during the course of radiotherapy starting on October 30, 2018. After 6 weeks, chest CT of the treated area revealed that the enlarged LNs in the retroperitoneum and mediastinum shrank or became loosened with an outcome partial response (PR) (Figure 14). The patient showed good tolerance, and no side effects except for bone marrow suppression were observed during the treatment.

#### Case 2

**Figure 15:**
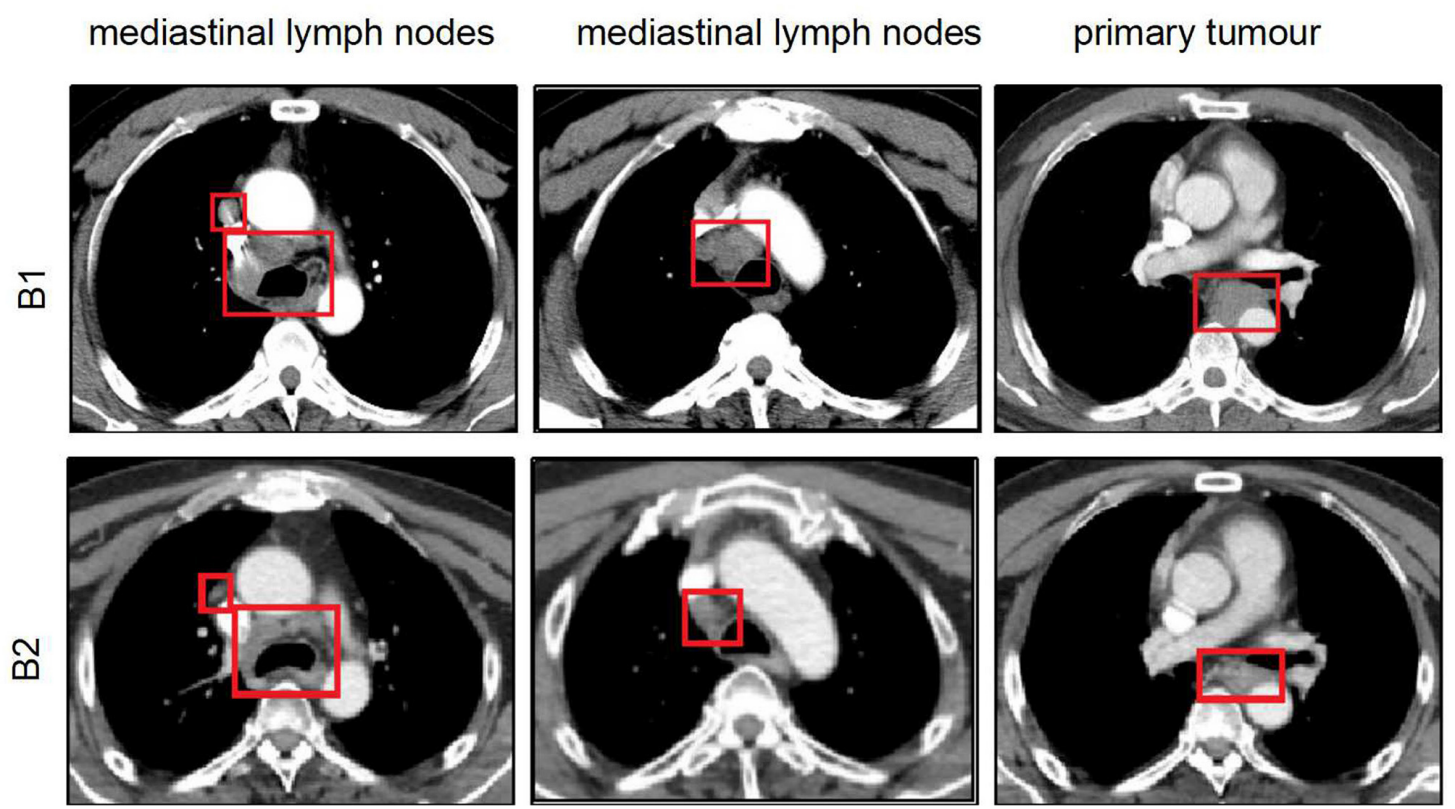
Comparison of chest CT scan between before and after treatment for the second patient. (B1) Chest CT showing that thickened esophageal wall and the enlarged lymph nodes in the mediastinum with an outcome of PD after patient underwent four cycles of chemotherapy. (B2) Chest CT scan representing that the primary tumor mass reduced in size and the enlarged lymph nodes in the mediastinum shrank or became loosened with an outcome of PR after anlotinib combined with radiotherapy and two cycles of chemotherapy. CT, computed tomography; PD, progressive disease; PR, partial progressive disease.

A 50-years-old man was diagnosed with ESCC with a pathological stage of IV (cT2N3M1) on April 9, 2019. This patient underwent two cycles of chemotherapy (120 mg of docetaxel, D1; 60 mg of nedaplatin, D1–2) on April 19 and May 9, 2019. Beginning on May 28, the patient was administered three cycles of docetaxel (120 mg, D1) in combination with nedaplatin (60 mg, D1–2) and tegafur (50 mg, D1–4). Chest CT scan on July 8, 2019, revealed an outcome of progressive disease (PD) (Figure 15). Then, the patient submitted written informed consent, and anlotinib (12 mg, D1–14, Q3W) was added to two cycles of chemotherapy (120 mg of docetaxel, D1; 60 mg of nedaplatin, D1–2; 50 mg of tegafur, D1–14) beginning on July 9, 2019. After combined treatment with the chemotherapeutic drugs and anlotinib, the patient was given radiotherapy with 2-Gy fractions once daily for 5 days per week to a total dose of 50 Gy beginning on August 19, 2019. After 6 weeks, chest CT of the treated area revealed that the primary tumor mass reduced in size and the enlarged LNs in the mediastinum shrank or became loosened with an outcome of PR (Figure 15). The patient showed good tolerance, and no serious side effects except for bone marrow suppression were observed during the treatment.

## Discussion

ESCC is a serious malignancy with a high mortality rate and poor prognosis in China, accounting for more than 95% of cases of EC (1, 2). Definitive radiotherapy or chemoradiotherapy followed by surgery has been used for the clinical treatment of locally advanced ESCC (17, 18). However, the 5-years survival rate remains unacceptably low. Therefore, studies aiming to identify effective therapeutic strategies that increase survival rates in ESCC are urgently needed.

Targeted drugs directed against molecules involved in the pathogenesis and progression of cancer have achieved successful clinical outcomes in other cancers. Genomic profiling using next-generation sequencing (NGS) has been conducted and has identified some candidate therapeutic targets for ESCC (19–21). However, clinical studies have shown that effective single-target drugs for ESCC are still lacking because of the complexity of signaling networks. Therefore, we proposed that multitarget drugs such as anlotinib may exhibit a better clinical activity in ESCC.

PDX models have recently emerged as ideal tools for the development of antitumor agents. In our study, we introduced this type of model to evaluate the efficacy and tolerability of anlotinib for ESCC treatment. This type of xenograft model has been reported in previous studies. For example, Jiang et al. established 26 ESCC patient tumor-derived xenografts and found that the pathological characteristics of the P3 xenografts, which expressed CK5/6, P40, and P63, were consistent with those of the original patient samples (22). Additionally, Zhou et al. reported that 40 PDX models are established from 188 esophageal carcinoma samples, and all consecutive generations of P3 and P4 cells maintained the same histology as the primary tumors (23). We first successfully established PDX models from three patients with ESCC. Because the experimental techniques used to establish the PDX models were well-established, only H&E staining and P63 expression were used for validation in our study. The validation assays demonstrated that the model histology remained similar between the passages and the original ESCC patient samples. Most importantly, H&E staining revealed interstitial tissues in addition to tumor cells; this finding benefited the development of our study and verified the reliability of the experimental results, because anlotinib targets the vascular system in malignancies. We also found that all tumor masses in control group exhibited a low microvessel density (MVD), indicating that angiogenesis plays an important role in ESCC development. These results were consistent with those of previous studies. For instance, Liu et al. demonstrated that a human umbilical vein endothelial cell (HUVEC) vaccine with anti-angiogenic effects on ESCC inhibited tumor growth in a humanized mouse model (24).

Because antiangiogenic drugs have usually been combined with other anticancer treatments in previous investigations, our study did not include a monotherapy group. Alternatively, radiotherapy is the main treatment for locally advanced ESCC. Therefore, we selected radiotherapy as the basis for designing our therapeutic regimens. We first investigated the effect of anlotinib on ESCC PDX tumor growth. The group treated with anlotinib combined with chemoradiotherapy exhibited significantly suppressed ESCC tumor growth and showed no significant loss of body weight than did the control group and the other three treatment groups. These data showed that anlotinib combined with chemoradiotherapy may exert a greater antitumor activity than either strategy alone and was relatively well-tolerated, without significant adverse reactions. To further explore the effect of anlotinib on tumor proliferation and apoptosis in ESCC, tissues from PDX mice were evaluated by TUNEL and immunohistochemical staining. The TUNEL results provided strong evidence that combined treatment with anlotinib and chemoradiotherapy, which induced the highest apoptosis rate in all groups, is likely to be a therapeutic option for ESCC patients. In the anlotinib treatment group, the vascular density was significantly reduced compared with that in the other groups. Collectively, these results demonstrated that anlotinib combined with chemoradiotherapy can inhibit tumor growth by increasing apoptosis and inhibiting angiogenesis. These results suggested that the inhibition of angiogenesis may play an important role in ESCC treatment in the future. However, esophageal bleeding should be noted in the clinical application of anlotinib.

In addition, we detected differences in the cytokine expression profiles between the anlotinib treatment group and other groups. Cytokines play an important role in innate immunity, apoptosis, angiogenesis, and cell growth and differentiation. They are involved in interactions between different cell types, the maintenance of homeostasis, and cellular responses to environmental conditions. More types of cytokines were released in the anlotinib treatment group than in the other groups in our study. The potential mechanism was that the body responded robustly to changes in the tumor, especially under an effective treatment regimen. Therefore, the cytokine profile indirectly reflects the therapeutic efficacy of anlotinib in ESCC.

Finally, we recruited two ESCC patients as case studies to further verify the therapeutic efficacy of anlotinib combined with chemoradiotherapy observed in PDXs. These two patients responded poorly to the initial tumor therapy regimens and had extensive LN metastasis. A previous study demonstrated that patients with ESCC who have extensive LN involvement have poor prognoses after conventional chemotherapy and radiotherapy. Zhao et al. showed that the 5-years survival rates were 43.6% for ESCC patients who received definitive (chemo)radiotherapy in the N0 group and 29.3% for those in the N+ group (*P* = 0.001) (24). Chen et al. reported that the PFS and OS times in the LN metastasis group were significantly shorter than those in the N0 metastasis group (9.8 vs. 5.9 months, *P* < 0.001; 18.2 vs. 9.7 months, *P* = 0.001, respectively), indicating that LN metastasis is an independent factor of poor prognosis in patients with locally advanced inoperable thoracic ESCC who have undergone concurrent chemoradiotherapy (25). In addition, a randomized multicenter study demonstrated that persistent pathological LN metastasis after neoadjuvant chemoradiotherapy (NCRT) plus surgery is a strong factor of poor prognosis in ESCC (26). The patients in our study who had extensive LN involvement were similar to those in the above literature reports. However, combination therapy with antiangiogenic drugs and chemoradiotherapy is non-standard treatment for ESCC patients; hence, only two patients are presented in our study. The first patient exhibited enlarged LNs soon after neoadjuvant chemotherapy and surgery. Therefore, this patient was given combination therapy with anlotinib and chemoradiotherapy with full informed consent. The second patient exhibited an outcome of PD with continued widespread LN enlargement after four cycles of chemotherapy. Considering the limited effect of chemotherapy, this patient submitted written informed consent and was given combination therapy with anlotinib and chemoradiotherapy. The two patients had similar clinical characteristics; that is, the conventional chemotherapeutic regimen showed poor efficacy before anlotinib intervention. Six weeks after combination therapy, both patients showed remarkable clinical benefits. Chest CT scans revealed that the primary tumor mass reduced in size and the enlarged LNs shrank or became loosened with an outcome of PR. Moreover, no esophageal bleeding, which we were concerned about, occurred during the treatment. In conclusion, the ideal anticancer effect of anlotinib combined with chemoradiotherapy observed in clinical patients was consistent with the results observed in PDX models, and no serious side effects were observed during the treatment, indicating that combination therapy with anlotinib and chemoradiotherapy may be an effective treatment regimen for patients with ESCC. However, adverse effects of the combination therapy need further confirmation in randomized clinical trials involving more patients before this approach can be used widely, although no serious side effects were observed in our study, possibly owing to the relatively small sample size. In addition, the combination approach and opportunity for anlotinib intervention, as well as the dosage of radiation used in the combination therapy, differed between the two patients. Hence, to achieve a better curative effect with fewer adverse reactions in ESCC patients, the optimal combination regimen needs to be defined in further clinical studies.

The limitations of our study should be emphasized. First, owing to the limitation of the surgical specimens and the low overall transplantation rate, the number of established PDX models was small. In addition, the selection of patients who accepted the therapeutic regimen of anlotinib combined with chemoradiotherapy in the clinic was difficult; therefore, only two case studies were presented herein. Finally, the molecular mechanisms of anlotinib combined with chemoradiotherapy need further exploration via basic experiments.

In conclusion, we successfully established ESCC patient tumor-derived xenografts that maintained the pathological characteristics of the patients' tumors. Our results in these models and clinical cases revealed the enhanced antitumor effect of adding anlotinib to chemoradiotherapy as well as the good tolerability of this regimen without significant adverse reactions, suggesting that combination therapy with anlotinib and chemoradiotherapy may be an effective regimen for the treatment of advanced ESCC.

## Data Availability Statement

The datasets generated for this study are available on request to the corresponding author.

## Ethics Statement

The studies involving human participants were reviewed, approved and supervised by the institutional research ethics board of the Shandong Tumor Hospital Ethics Committee (SDTHEC201703014). The patients/participants provided their written informed consent to participate in this study. Written informed consent for the use of the tissue samples and the publication of any potentially identifiable images or data was obtained from every patient before enrollment in the study. The animal study was reviewed and approved by the ethics committee of Shandong Cancer Hospital affiliated to Shandong Universitiy, and followed internationally recognized ARRIVE (Animal Research: Reporting of in vivo Experiments) guidelines.

## Author Contributions

ZL, JL, and JS designed and conceived the study materials and contributed to the data collection and assembly, and manuscript drafting. YZ and JW participated in analyzing and interpreting data and in revising the content. All authors read and approved the final manuscript.

## Conflict of Interest

The authors declare that the research was conducted in the absence of any commercial or financial relationships that could be construed as a potential conflict of interest.
