# Corrigendum: Anlotinib Combined With Chemoradiotherapy Exhibits Significant Therapeutic Efficacy in Esophageal Squamous Cell Carcinoma

**DOI:** 10.3389/fonc.2020.583499

**Published:** 2020-11-20

**Authors:** Jingzhen Shi, Yingjie Zhang, Jinzhi Wang, Jianbin Li, Zhenxiang Li

**Affiliations:** ^1^School of Medicine, Shandong University, Jinan, China; ^2^Shandong Cancer Hospital Affiliated to Shandong University, Jinan, China; ^3^Department of Radiation Oncology, Shandong Cancer Hospital and Institute, Shandong First Medical University and Shandong Academy of Medical Sciences, Jinan, China

**Keywords:** patient-derived xenograft, esophageal squamous cell carcinoma, anlotinib, chemoradiotherapy, anti-angiogenesis

In the original article, there was a mistake in Figure 1 and Table 2 as published. Duplicated panels were contained in Figure 1 due to author error. The authors provided a modified Figure 1 during the review process. The data of the pre-experiment were misplaced in Table 2 and submitted after the proofs due to author error. The corrected Figure 1 and Table 2 appear below.

**Figure 1:**
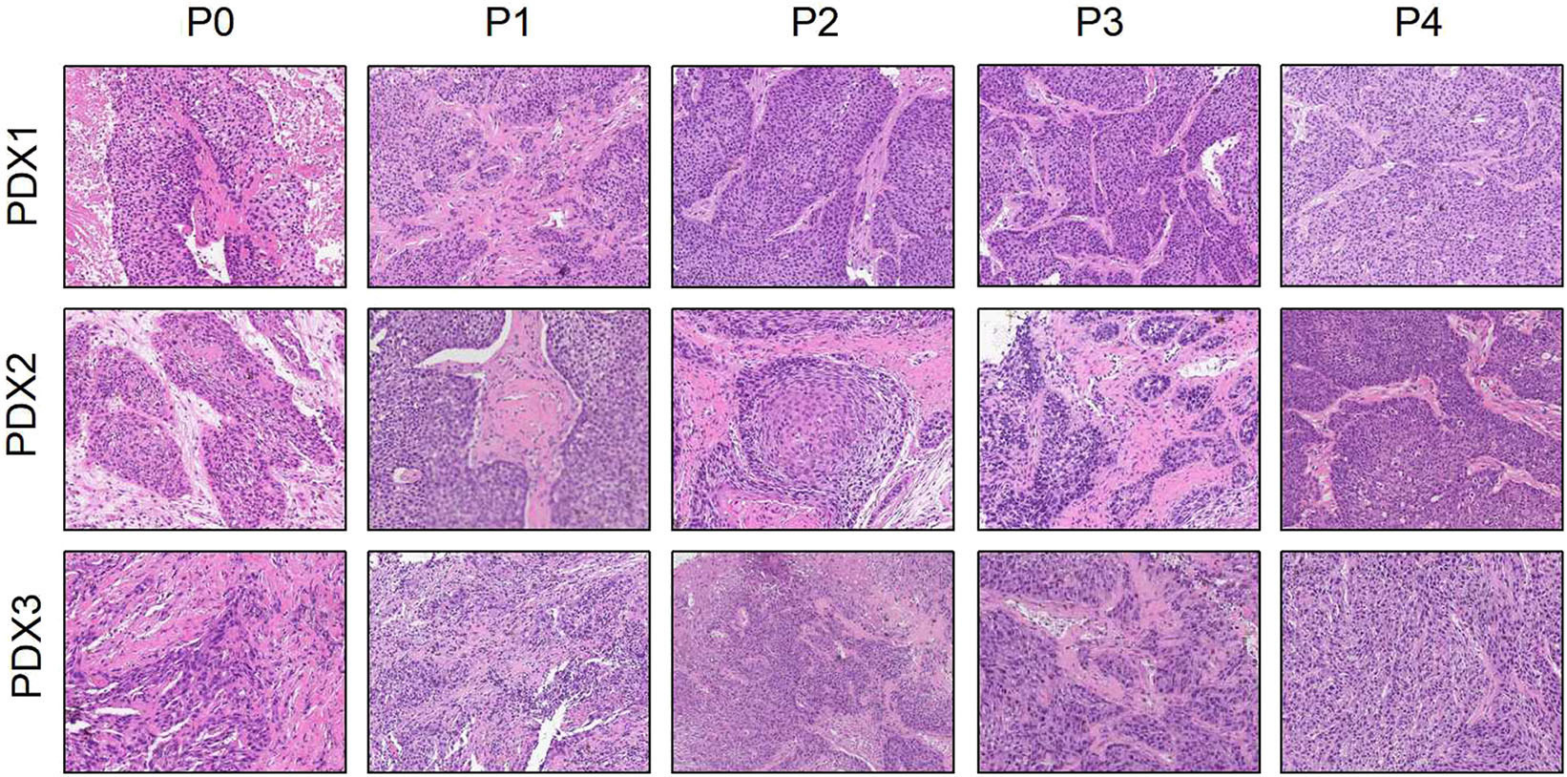
Histological section (H&E) presented that the esophageal patient tissue (P0) was consistent with the series passages (P1, P2, P3, and P4) in the SCID mice (×200).

**Table 2:** The detailed data of tumor volume and body weight.

**No**.	**Tumor volume (mm^3^)**	**Control group**	**RT**	**RT+DDP**	**RT+Anlotinib**	**RT+DDP+Anlotinib**
PDX1	Initial	266.64 ± 37.05	243.4 ± 31.38	252.61 ± 60.13	260.90 ± 43.67	267.24 ± 51.13
	Final	343.51 ± 29.22	188.54 ± 40.04	150.31 ± 15.87	144.29 ± 15.05	121.02 ± 25.72
	Changing	77.06 ± 32.90	−54.95 ± 39.63	−102.31 ± 46.44	−116.61 ± 45.17	−146.23 ± 48.59
PDX2	Initial	187.52 ± 62.04	171.5 ± 31.30	185.45 ± 44.30	178.06 ± 40.02	181.31 ± 15.67
	Final	246.07 ± 109.69	116.3 ± 25.17	133.11 ± 53.32	119.17 ± 30.60	71.79 ± 5.88
	Changing	58.55 ± 64.42	−55.11 ± 22.68	−52.34 ± 55.52	−58.89 ± 38.21	−109.52 ± 18.05
PDX3	Initial	267.39 ± 28.18	265.4 ± 57.22	267.33 ± 53.02	256.01 ± 30.76	262.82 ± 29.66
	Final	648.68 ± 222.59	130.7 ± 57.33	161.87 ± 45.37	124.46 ± 38.87	70.89 ± 15.46
	Changing	381.33 ± 251.804	−134 ± 17.26	−105.46 ± 13.90	−131.54 ± 35.45	−191.93 ± 42.70
**No**.	**Body weight (g)**	**Control group**	**RT**	**RT+DDP**	**RT+Anlotinib**	**RT+DDP+Anlotinib**
PDX1	Initial	25.92 ± 22.15	24.85 ± 2.06	26.3 ± 41.20	24.7 ± 81.34	24.29 ± 1.57
	Final	25.99 ± 0.50	24.77 ± 2.30	25.9 ± 62.02	23.9 ± 12.47	23.45 ± 2.27
	Changing	0.06 ± 2.53	−0.08 ± 0.73	−0.88 ± 1.84	−0.87 ± 2.56	−0.83 ± 1.39
PDX2	Initial	22.68 ± 1.79	22.25 ± 0.84	22.66 ± 1.08	22.32 ± 1.48	22.71 ± 2.71
	Final	24.07 ± 0.77	23.35 ± 1.82	23.03 ± 1.01	22.05 ± 1.87	23.29 ± 1.47
	Changing	1.60 ± 1.23	1.10 ± 1.21	0.37 ± 0.84	−0.27 ± 1.17	0.57 ± 1.46
PDX3	initial	22.00 ± 2.54	21.23 ± 2.77	20.86 ± 2.92	21.99 ± 2.70	22.05 ± 2.58
	Final	24.21 ± 1.31	22.10 ± 1.30	22.86 ± 0.97	22.84 ± 1.07	22.69 ± 0.95
	Changing	2.21 ± 2.33	1.16 ± 1.72	2.00 ± 3.17	0.85 ± 3.15	0.64 ± 3.11

*RT, radiation therapy; DDP, cisplatin*.

The authors apologize for this error and state that this does not change the scientific conclusions of the article in any way. The original article has been updated.

